# Multi-Channel Based Image Processing Scheme for Pneumonia Identification

**DOI:** 10.3390/diagnostics12020325

**Published:** 2022-01-27

**Authors:** Grace Ugochi Nneji, Jingye Cai, Jianhua Deng, Happy Nkanta Monday, Edidiong Christopher James, Chiagoziem Chima Ukwuoma

**Affiliations:** 1 School of Information and Software Engineering, University of Electronic Science and Technology of China, Chengdu 611731, China; ugochinneji@std.uestc.edu.cn (G.U.N.); jianhua.deng@uestc.edu.cn (J.D.); edianajames@yahoo.com (E.C.J.); ukwuoma@std.uestc.edu.cn (C.C.U.); 2 School of Computer Science and Engineering, University of Electronic Science and Technology of China, Chengdu 611731, China; mh.nkanta@std.uestc.edu.cn

**Keywords:** local binary pattern (LBP), contrast limited adaptive histogram equalization (CLAHE), contrast enhanced canny edge detection (CECED), deep learning, image identification, pneumonia disease, COVID-19

## Abstract

Pneumonia is a prevalent severe respiratory infection that affects the distal and alveoli airways. Across the globe, it is a serious public health issue that has caused high mortality rate of children below five years old and the aged citizens who must have had previous chronic-related ailment. Pneumonia can be caused by a wide range of microorganisms, including virus, fungus, bacteria, which varies greatly across the globe. The spread of the ailment has gained computer-aided diagnosis (CAD) attention. This paper presents a multi-channel-based image processing scheme to automatically extract features and identify pneumonia from chest X-ray images. The proposed approach intends to address the problem of low quality and identify pneumonia in CXR images. Three channels of CXR images, namely, the Local Binary Pattern (LBP), Contrast Enhanced Canny Edge Detection (CECED), and Contrast Limited Adaptive Histogram Equalization (CLAHE) CXR images are processed by deep neural networks. CXR-related features of LBP images are extracted using shallow CNN, features of the CLAHE CXR images are extracted by pre-trained inception-V3, whereas the features of CECED CXR images are extracted using pre-trained MobileNet-V3. The final feature weights of the three channels are concatenated and softmax classification is utilized to determine the final identification result. The proposed network can accurately classify pneumonia according to the experimental result. The proposed method tested on publicly available dataset reports accuracy of 98.3%, sensitivity of 98.9%, and specificity of 99.2%. Compared with the single models and the state-of-the-art models, our proposed network achieves comparable performance.

## 1. Introduction

Pneumonia is an infectious lung illness in humans that affects one or both lungs and is caused by fungus, bacteria, and viruses, among other microorganisms. Pneumonia occurs as a result of pathogen-caused inflammation [1], which causes the alveoli in the lungs to fill up with pus or fluid, reducing oxygen (O${}_{2}$) and carbon-dioxide (CO${}_{2}$) exchange between the lungs and blood, making it difficult for the infected person to breathe. Other causes of pneumonia are food aspiration and chemical exposure. Furthermore, patients with cancer, HIV/AIDS, hepatic disease, diabetes, cardiovascular diseases, chronic respiratory diseases, and other comorbidities, are vulnerable to pneumonia [1,2,3]. As the result of the inability to identify this lung illness at an early stage, children below the age of five years and aged people are readily transmitted with this illness.

There are several methods to diagnose pneumonia, which include blood test, pulse oximetry, bronchoscopy, sputum test, pleural fluid culture, chest X-ray, magnetic resonance imaging, and CT scans. The blood tests are carried out to confirm an illness and to attempt to figure out the organism caused by it. However, this method is not always feasible to make an accurate identification. Pulse oximetry test determines how much oxygen is in the blood. Pneumonia may make it difficult for the lungs to get enough oxygen into the circulatory system. Another method of pneumonia diagnose is the sputum test which demands for a patient’s deep cough, then the sample of fluid from the lungs (sputum) is obtained and tested to assist determine the source of the illness. A culture method called pleural fluid culture requires a collection of a fluid sample from the pleural region by inserting a needle between the patient’s ribs and testing it to help diagnose the kind of infection. Chest X-ray is used to diagnose pneumonia and evaluate the degree and location of the illness. However, it cannot tell which bacterium caused the pneumonia. Finally, for a clearer view of the lungs, CT scans are highly recommended by the medical practitioners. For older citizens above 65 age of years with serious symptoms and health condition they are advised to undergo imaging examination such as CT scans [4,5]. In most cases, the causing organism determines how pneumonia is treated. Anti-fungal treatments are used to treat fungus pneumonia whereas antiviral medications are used to treat viral pneumonia including influenza, SARS, and MERS, and antibiotics are used to treat bacterial pneumonia [6]. Despite the presence of pneumonia, the diagnosis is always dependent on the doctor’s knowledge and experience. Howbeit, as the number of infected patients’ increases, it becomes a difficult task for radiologists to access the screening process within a constrained time; therefore, there is a need for an AI diagnostic system for the classification of pneumonia.

Deep learning (DL) algorithms have ascertained greater performance in the classification and detection of pneumonia-related ailment and provided high accuracy rate when compared to other methods. Emphatically, DL can identify hidden features of images which could never be detected by medical professionals. With regards to DL, the convolutional neural network (CNN) is the most often utilized DL technique in the medical system due to its capacity in feature extraction and training in differentiating between multiple classes. Transfer learning (TL) technique has made it simpler to retrain deep neural networks fast and accurately on different datasets [7,8,9]. With the introduction of wavelet in CNN, Happy et al. [10] proposed a CNN model integrated with wavelet multi-resolution analysis to effectively classify COVID-19 pneumonia from radiographs. Interestingly, Happy et al. [11] presented a case study to evaluate the capability of multi-resolution analysis in diagnosing COVID-19 pneumonia.

Several applications of computer vision are so complicated that they cannot be achieved using only one algorithm, which has necessitated the creation of models that combined two or more of the methods evaluated. Since models are chosen based on the issue’s requirements and features extraction, weighted fusion technique incorporates more than one model to address this issue. This technique was created to help single models overcome their flaws and solidify their strengths by aggregating the features extracted from single model in a weighted manner. This method reduces generalization error and minimizes prediction variance [12]. Thus, the goal of this study is to analyze the performance of weight fusing deep neural network models for multi-class of pneumonia. The following are the main contributions of our paper:

- We preprocess our CXR images into three forms, namely: LBP, CLAHE, and CECED.
- Each preprocessed image features are extracted individually, that is the LBP CXR features are extracted using the shallow CNN, the CECED CXR features are extracted using the MobileNet-V3 and the CLAHE CXR feature images are extracted using the Inception v3.
- The feature vectors of these preprocessed CXR images are weighted fused for a robust prediction result.
- We evaluate the performance of each of the single models and our proposed model in this study.

The remaining part of this paper is broken down as follows; the related works on pneumonia disease are discussed in Section 2. Section 3 explains how we came up with our proposed framework. The experimental results and evaluation of our model are presented in Section 4. Our discussion is presented in Section 5. Finally, Section 6 presents the conclusion.

## 2. Related Works

In recent years, publications of the some of the state-of-the-art models used the deep learning (DL) approach for the automatic classification and detection of pneumonia from X-ray images. This section reviews and examines the most up-to-date approaches for detecting and classifying pneumonia with DL.

Various investigations and research studies based on artificial intelligence and deep learning have been conducted in the area of disease diagnosis using medical imagery such as chest X-rays (CXR), ultrasound scans, computed tomography (CT) scans, MRI scans, and so on. Deep learning is perhaps the most widely used and reliable medical imaging approach available. It is a quick and accurate way for diagnosing variety of ailments. There are models that have been explicitly trained to classify different categories of specific ailment based on the disease type. These models have proven to achieve satisfactory results in the medical science utilizing image analysis for the detection of cardiac abnormalities, tumors, cancer, and a variety of different applications.

Deep learning has been used to distinguish between scan images of COVID-19-infected and non-infected patients as proposed by Shah et al. [13] using a self-developed framework called CTnet-10, hence, achieving an accuracy of 82.1%. However, to further enhance the accuracy, different pretrained models were introduced to the CT scan dataset and VGG-19 attain a better performance of 94.5%.

Deep neural network has the ability to improve the output of instruments utilized in pharmaceutical processes for analytical technology. Maruthamuthu et al. [14] developed a technique for identifying the contamination of micro-organisms using DL strategies based on Raman spectroscopy. The categorization of numerous sample consisting of micro-organisms and bacteria mixed with Chinese Hamster Ovary cells achieved an accuracy of 95% to 100% using a convolutional neural network (CNN).

Looking at a situation of very large margin of unlabeled dataset, Maruthamuthu et al. [15], demonstrate the superiority of a data-driven strategy over data analytics and machine learning technique. The data analytics strategy, however, has the potential to improve the development of efficient proteins and affinity ligands, affecting the downstream refinement and processing of mAb products, according to the authors. The authors propose a deep neural network approach, which is also called a black box for the construction of soft sensors, which uses accumulated past data from process operation that is not present in a mechanistic orphenomenological approach. The data are incorporated to get apolynomial expression to generate product as a function of the inputs such as cell mass, substrate concentration, and initial product (streptokinase). Given enough dataset collected from various sources under different condition, the accurate data fit with automated error correction generated factor is utilized to compute the major outputs such as the number of workable cells and extent of product as a result of the marginal calibrated input parameters such as inoculum and substrate. Even though the molecular relationship of these variables to cell metabolism is uncertain, yet the deep learning model accounts for the impacts of other conditions such as excitation, individual critical media properties, mixing rate, and variations in metabolic concentrations.

An application of the Inception-v3 model is used by Kermany et al. [16] to classify different types of pneumonia infections in pediatric patients. This method extracts features and utilizes the area under curve (AUC) as a metric to categorize bacterial and viral pneumonia. Santosh et al. [17] suggested a method for detecting pulmonary problems by comparing the symmetrical shape of the CXR lung region. Roux et al. [18] sought to determine the prevalence, intensity, and adverse outcomes for pneumonia in infants during their first year of life in a South African cohort study. They set up pneumonia monitoring systems and recorded outpatient pneumonia and pneumonia that required hospitalization. In addition, combined Poisson regression was used to calculate pneumonia incidence rate ratios.

Table 1 gives a summary of the related works with the following observations:

- Several DL techniques are thoroughly implemented for the classification task.
- More so, researchers in [27,28,31,32] use more than five DL techniques to evaluate the performance.
- The popular medical mode of imaging for the classification and detection of pneumonia-related ailment is the chest X-ray.
- The publishers focus on either binary classification or multi-classification, although just a few considered the multi-classification task.
- Some evaluation metrics such as accuracy, sensitivity, and specificity were utilized to evaluate the efficacy of DL models approaches whereas precision, recall, and/or F1-score metrics were used in [25,26] to evaluate the model performance.

However, there was no account where low quality was considered as a challenge for better classification performance of pneumonia. Therefore, we put this into consideration by preprocessing our images into LBP, CECED, and CLAHE for multi-channel weighted fusion technique using neural network for the identification of pneumonia. Additionally, we carried out a study to compare the performance of each single model and the weighted fusion model.

## 3. Materials and Methods

The methodology employed in this study is detailed in this section. Data collection, image preprocessing, and the transfer learning networks for feature extraction and classification are the three phases of the proposed method. The procedure of the proposed method in this paper is presented in the subsequent subsections.

### 3.1. Datasets

This study utilized the pneumonia dataset from two public domain. The first dataset contains 3029 scans of bacterial pneumonia, 8851 scans of healthy patients and 2983 scans of viral pneumonia gotten from the Kaggle database of RSNA [33]. The second dataset is taken from the author in [34] which consist of 3616 CXR images of patients diagnosed with COVID-19. In all, we only considered 1000 CXR images each for the four classes (COVID-19, virus, bacteria, and normal), as explicitly illustrated in Table 2.

### 3.2. Data Preprocessing

It is well known that data quality can be affected by factors such as noise, resolution and artifacts, hence, using such data directly in an algorithm may result to inaccurate outcomes. Data preprocessing phase could help in eliminating or reducing the noise and increase the data quality, thereby improving model performance. In this study, we have use the Local Binary Pattern (LBP), Contrast-Enhanced Canny Edge Detection (CECED), and Contrast Limited Adaptive Histogram Equalization (CLAHE) for our data preprocessing.

#### 3.2.1. Local Binary Pattern (LBP) Images

**Figure 1:**
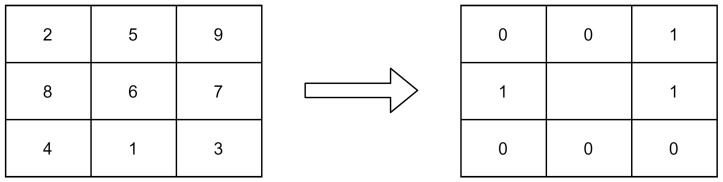
LBP coding and calculation illustration.

**Figure 2:**
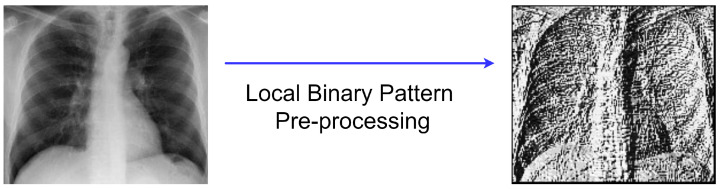
LBP pre-processing.

A descriptor called LBP is often used to collect textural information about a specific object. Coding LBP pixel is determined by comparing its value to that of neighboring pixels [13]. At the left portion of Figure 1 depicts all the values of the pixel in a local region, whereas the right part depicts the binary pixel of the LBP coding while concentrating on the left pixel. The pixel value of LBP can be computed after it has been efficiently encoded using LBP coding. Equation (1) gives the mathematical expression of LBP.

(1) $$LBP=\sum_{n=1}^{N}S\left(g_{n}*g_{c}\right)*2^{n}$$

where $S(g_{n}-g_{c})$ denotes the indicator function for the threshold binary value obtained from the subtraction of the neighbor pixel value from center pixel value. N is the total number of pixel value surrounding the center pixel value for the 3 × 3 window size since LBP considers 9 pixels at a time. $g_{c}$ represents the center pixel value and $g_{n}$ denotes the neighbor pixels values. After the thresholding, the pixel’s value greater or equal to zero would be “1” and the pixel’s value less than zero would be “0”. Figure 1 illustrates the calculation of the pneumonia LBP CXR image and Figure 2 presents the preprocessing of LBP.

#### 3.2.2. CLAHE Images

**Figure 3:**
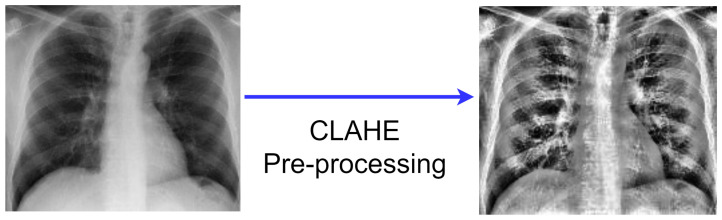
CLAHE Pre-processing.

CLAHE was used in this study to improve the image’s contrast and features by making abnormalities more noticeable. Among the histogram equalization family, Contrast Limited Adaptive Histogram Equalization (CLAHE) is more natural in appearance and useful in the reduction of noise amplification, and we have investigated CLAHE and applied it to our dataset, as shown in Figure 3. A full explanation of the CLAHE approach is given below to demonstrate its effectiveness:

- The generation of the image transformation using the bin value of the histogram is the first stage of the CLAHE technique.
- Following that, using clip boundary, the contrast is confined to a binary count from 0 to 1. Before the image segment is processed, the clip boundary is added to the image.
- To prevent mapping background areas to gray scale, a specific bin value from the histogram region is used to create the entire image region. Clip boundary is used with the help of histogram clip to obtain better mapping.
- Finally, the finished CLAHE image is created by computing the image’s regions, then extracting, mapping, and interpolating all of the image pixels to get the most out of the image.

#### 3.2.3. CECED Images

**Figure 4:**
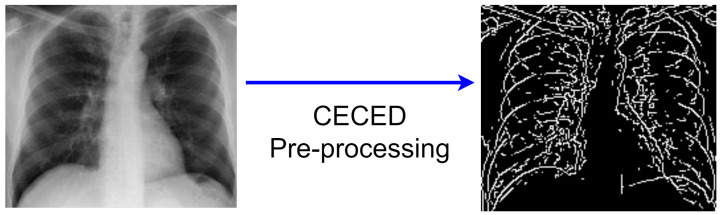
CECED Pre-processing.

As shown in Figure 4, edges are made up of crucial and relevant particular information and features. By employing an edge detector on an image, the amount of data that has to be processed can be reduced, and the information that is deemed less significant can be filtered. The CEED-Canny strategy combines the local morphological contrast enhancement and the Canny edge detection technology used in [24]. The steps are as follows:

- Collection of the original pixel’s value, as well as the local minimum and maximum;
- The image’s morphological contrast is increased;
- To reduce noise, Gaussian smoothing is applied;
- The intensity gradient of the image is determined;
- A non-maximum suppression method is utilized;
- The hysteresis thresholding technique is adopted.

### 3.3. Feature Extraction

This article uses three CNN architectures: a shallow CNN, pretrained MobileNet-V3, and Inception-V3 as feature extractors with their layers trained and/or frozen. To prevent the model from overfitting, dropout, and regularizer are applied.

#### 3.3.1. Features Extraction from LBP Images

**Figure 5:**
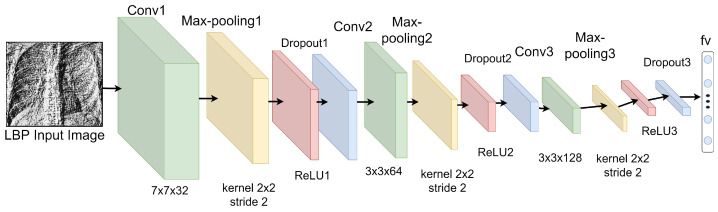
Shallow CNN structure applied for the feature extraction of LBP CXR images.

For an automatic extraction of pneumonia-related features from LBP CXR images, we develop a shallow CNN model. Shallow CNN model simply means a lightweight convolutional neural network constructed from scratch to process LBP CXR images. In our study, we constructed a three-layer convolutional neural network which we called shallow CNN, as illustrated in Figure 5. It has an input layer, three convolution (“C”) and subsequent sub-sampling (“S”) layers, and a feature vector (“fv”) layer, as shown in Figure 5. The first convolution layer (“C1”) employs 32 filters and 7×7 convolution kernel that focus on the detailed information of possible indicators of pneumonia. This layer is followed by a sub-sampling layer (“S1”), which reduces the image to half its original size using optional max-pooling (with kernel size 2×2).

To map the previous layer, a new convolution layer (“C2”) conducts 64 convolutions with 3×3 kernel, followed by another sub-sampling layer (“S2”) with 2×2 kernel. Lastly, 128 convolution layer (“C3”) with 3×3 kernel is applied and followed by a max-pooling (with kernel size 2×2). Finally, we arrived at a feature vector of 512-neuron after the output is passed to the fully connected layer (“fv”). Adding “ReLU” activation after sub-sampling layers “S1” and “S2” guarantees the capacity to handle nonlinear data. Over-fitting can be avoided by utilizing the “dropout” operation [31] between the “S” layers (“S1” and “S2”) and the “fv” layer (parameter was set to 0.5).

#### 3.3.2. Features Extraction from CECED CXR Images

**Figure 6:**
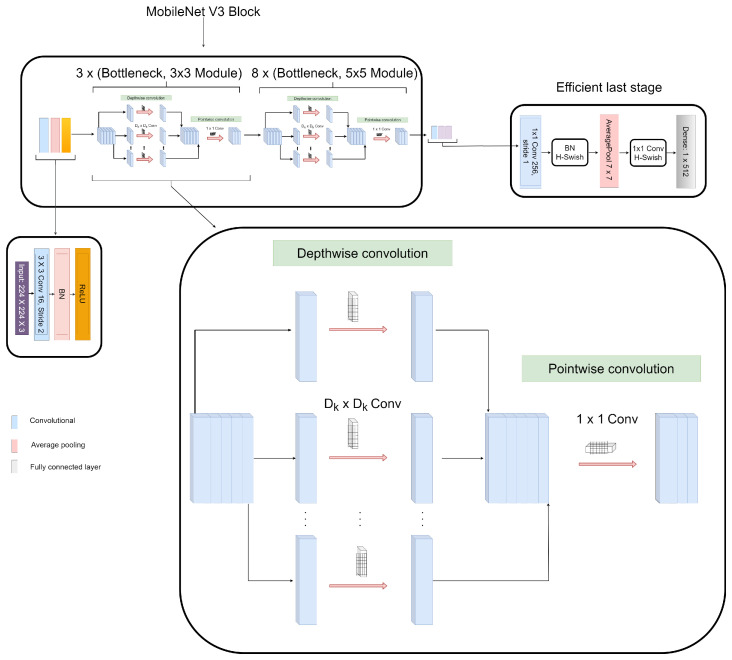
Framework of the pretrained MobileNet-V3 utilized for the features extraction of CECED CXR images.

We utilized the MobileNet-V3 network in this extraction phase because of its efficient performance in image classification and fast convergence. Table 3 depicts the parameters of the network. The model has an input size of 224×224 input image. It uses depth-wise separable convolutions, which applies two 1D convolutions with two kernels, rather than a single 2D convolution. This allows for a smaller and more efficient model with less memory and training parameters. The model is divided into two blocks: the residual block with a stride of 1 and the shrinking block with a stride of 2. Each block is made up of three layers: a 1×1 convolution with ReLU6, a depth-wise convolution, and another 1×1 convolution with different non-linearity. A few changes were made to our network to simplify it by replacing the dense layer with a dimension of 1×512, as shown in Figure 6.

#### 3.3.3. Features Extraction from CLAHE CXR Images

The high performance of Inception-V3 [25] is due to a variety of network connection approaches, including batch normalization, using MLP convolutional layers to replace linear convolutional layers, and factorizing convolutions with larger kernel sizes. These methods significantly reduce the number of network parameters as well as the computational cost of the network, allowing it to be built considerably deeper and with more nonlinear expressive capacity than typical CNN models.

**Figure 7:**
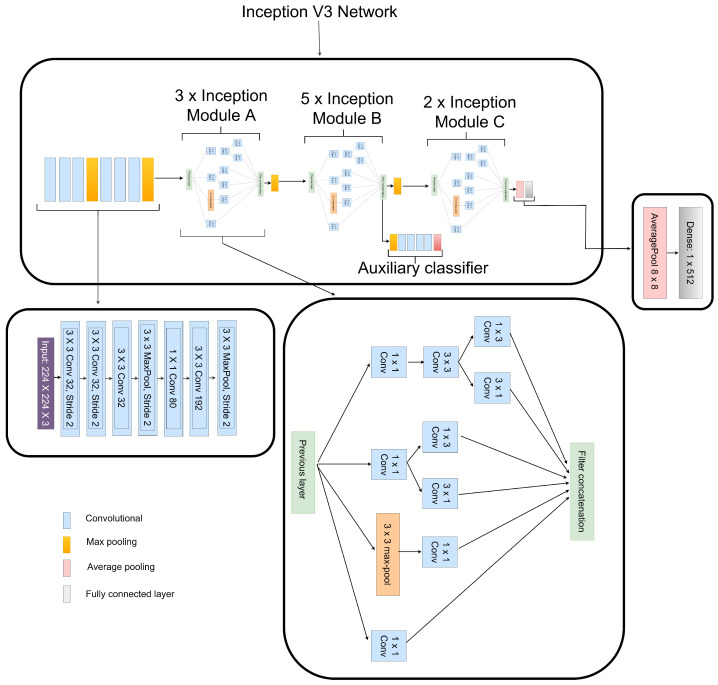
Framework of the pretrained Inception-V3 utilized for the features extraction of CLAHE CXR images.

The Inception-V3 model proposed in this paper was previously trained over the huge dataset ImageNet, and then the knowledge gained from transfer learning was transferred to CXR dataset to perform pneumonia identification. All layers before the fully connected (FC) and softmax layers were frozen. The softmax layer is eliminated and a new dense layer is trained for extracting the deep-level features of the CXR images by continuously modifying the network’s parameters, resulting in a feature vector size of 1×512 as seen in Figure 7. Similar to the authors in [25], average pooling of 8×8 is employed instead of the traditional fully connected layer to flatten the feature vector as seen in Figure 7.

### 3.4. Weighted Fusion of the Different Output Channels

**Figure 8:**
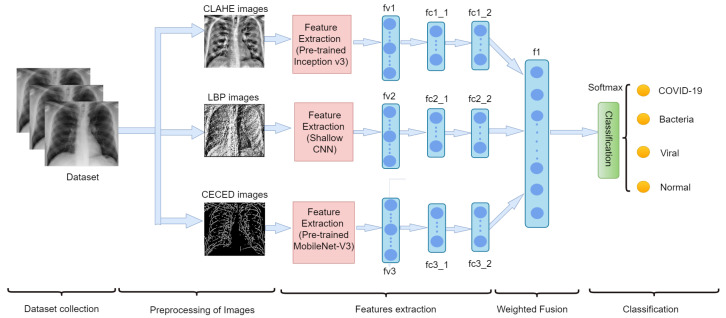
Our proposed multi-channel scheme for pneumonia identification.

According to the proposed model shown in Figure 8, shallow CNN is used to extract the feature vector (fv1) from the LBP CXR images, the CLAHE CXR images’ feature vector (fv2) is retrieved using a pretrained Inception-V3 approach and the pretrained MobileNet-V3 is utilized to extract feature vector (fv3) from CECED CXR images. After flattening, each feature vector is connected to two dense layers. The first dense layers for fv1, fv2, and fv3 are fc1_1, fc2_1, and fc3_1, respectively, while the second dense layers are fc1_2, fc2_2, and fc3_2, respectively, as shown in Figure 8. Using fc1_2 and fc2_2 and fc3_2, the network captures the distances between various lung properties and reveals them. Furthermore, fc1_2, fc2_2, and fc3_2 are fused in a weighted way into f1 to generate a fused vector. Softmax is used to classify pneumonia CXR images based on the fused feature vector. As a cost function, the categorical cross entropy is utilized, given in Equation (2)

(2) $$L_{loss}=-\sum_{k=1}^{n}t_{k}\;log\;p_{k}$$

where *n* represents the number of class labels, $p_{k}$ is the instances for the k-th classes and $t_{k}$ is the corresponding label The chance of event *k* occurring is $t_{k}$ meaning that the total sum of $t_{k}$ is 1, which implies that only one event is possible. The negative sign minimizes the loss when the distributions come closer to one other.

In the last layer of our proposed weighted model with categorical cross entropy loss function, the softmax function is utilized as a classifier to emphasize the highest values and suppress those that are far below the maximum value as well as normalizing the outputs to the total sum of 1, as illustrated in Equation (3)

(3) $$p_{k}=\frac{e^{tk}}{\sum_{j=1}^{n}\;e_{j}^{t}}$$

where $e^{tk}$ is the exponential term of the input vector, *n* is the number of class label, $p_{k}$ is the output vector of the softmax and $\sum_{j=1}^{n}\;e^{t_{j}}$ is the normalization term which divides the exponential term to produce a real value greater than 0.

## 4. Results

This section gives thorough explanations of (1) the experimental setup and configuration (2) the performance metrics utilized for the evaluation of the proposed framework; (3) the performance of individual networks, (4) the performance of weighted fusion technique, and (5) comparisons with other research models.

### 4.1. Experimental Setup and Configuration

Our proposed model is evaluated using the Keras framework with Python programming language using NVIDIA GTX 1080 GPU. A data split ratio of 80% to 20% for training and testing, respectively. All the data are resized for each single model. For the shallow CNN, our LBP input dataset are resized to 112×112, whereas for the pretrained MobileNet-V3 network, the CECED CXR input dataset are resized to 224×224 and lastly for the pre-trained inception-V3 network, the CLAHE CXR input dataset are resized as 299×299. Using Adam optimization, we utilized batch size of 32, learning rate of 0.0001, L2-regularizer, weight decay, dropout to reduce over-fitting in the model and epoch size of 40. We trained separately each of the the single model (shallow CNN, pre-trained MobileNet-V3 and pre-trained Inception-V3) and the concatenation of these single models using the fusion technique with the same dataset. More so, the output classes of 1000 in ImageNet were replaced with 4 classes in the final dense layer, representing viral, bacteria, normal, and COVID-19.

### 4.2. Performance Metrics

In evaluating the performance of the single and concatenated models, this paper used the evaluation metrics of accuracy, sensitivity, specificity, precision, and F1-score. The numerical expression for each metric is presented in Equations (4)–(8).

(4) $$\mathrm{Accuracy}=\frac{\mathrm{TP}+\mathrm{TN}}{\mathrm{TP}+\mathrm{TN}+\mathrm{FP}+\mathrm{FN}}$$

(5) $$\mathrm{Sensitivity}=\frac{\mathrm{TP}}{\mathrm{TP}+\mathrm{FN}}$$

(6) $$\mathrm{Specificity}=\frac{\mathrm{TN}}{\mathrm{TN}+\mathrm{FP}}$$

(7) $$\mathrm{Precision}=\frac{\mathrm{TP}}{\mathrm{TP}+\mathrm{FP}}$$

(8) $$F1-\mathrm{score}=2*\frac{\mathrm{Precision}*\mathrm{Recall}}{\mathrm{Precision}+\mathrm{Recall}}$$

TP, FP, FN, and TN represent true positive, false positive, false negative, and true negative, respectively.

### 4.3. Evaluation of the Single Model

We begin by presenting the accuracy, loss, ROC-AUC, and precision–recall curves produced by the various deep learning frameworks used in this article (Inception-V3, MobileNet-V3, and Shallow CNN). Then, using the metrics established in Equations (4)–(8), we evaluated the results of all designs to find the best method for classifying CXR images as bacteria, COVID-19, normal, and viral pneumonia as shown in Table 4. The ROC-AUC, precision–recall, accuracy, and loss curves, as well as the several models utilized in this study and their interpretation, are shown in the next section.

#### 4.3.1. Shallow CNN

It is worth noting that the shallow CNN achieved considerable performance. The results from Table 4 show that the shallow CNN obtained 90.9% accuracy, 92.3% sensitivity, and 93.1% specificity. In comparison to Inception-V3 and MobileNet-V3, shallow CNN achieved the least score across all the evaluation metrics. Furthermore, the shallow CNN obtained 91.2% precision and 92.7% F1-score.

#### 4.3.2. Pretrained MobileNet-V3

Considering the evaluation performance metrics for the single model of MobileNet-V3, this model obtained accuracy of 93.7%, sensitivity of 95.4%, specificity of 95.7%, precision of 94.3%, and F1-score of 95.5%. These results further show that the CECED preprocessing technique using the MobileNet-V3 performs better than the LBP shallow CNN technique.

#### 4.3.3. Pretrained Inception-V3

From the experimental results presented in Table 4, Inception-V3 shows satisfactory performance achieving accuracy of 95.6%, 94.9% sensitivity, and 96.2% specificity, precision of 95.8%, and F1-score of 95.3%. Compared to shallow CNN, this inception-V3 seems to perform slightly higher than shallow CNN across all the evaluation metrics, however, Inception-v3 achieves better performance on few evaluation metrics such as accuracy, specificity, and precision over the MobileNet-V3.

### 4.4. Evaluation of the Weighted Fusion

**Figure 9:**
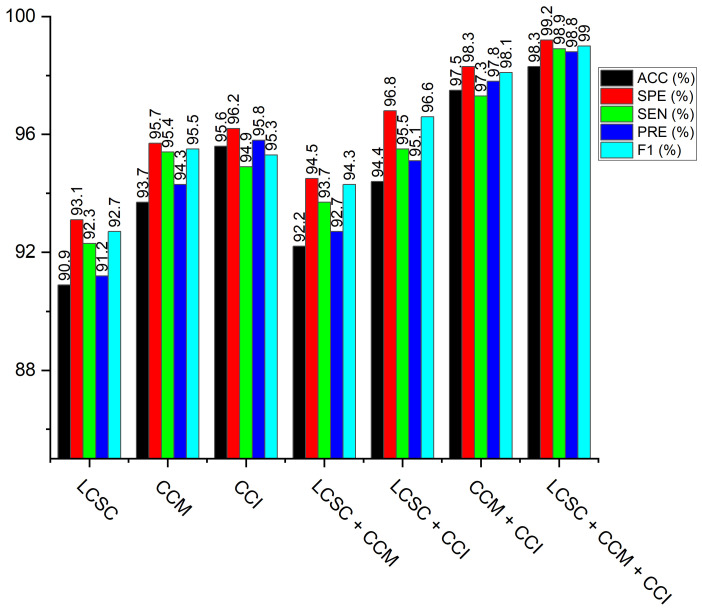
Performance of the proposed multi-channel in comparison with the single channels and dual channels across the different evaluation metrics.

On the pneumonia dataset, we executed various weighted fusion approaches to see the better performance for the identification of pneumonia. Our first investigation is the weighted fusion of LBP-channel shallow CNN and CECED-channel of MobileNet-V3 (LCSC + CCM). Secondly, we checkmate the fusion of the LBP-channel of shallow CNN and the CLAHE-channel of Inception-V3 (LCSC + CCI). The next analysis is the fusion of CECED-channel of MobileNet-V3, and CLAHE-channel of Inception-V3 (CCM + CCI)) and the last weighted-manner approach is the fusion of LBP-channel of shallow CNN, CLAHE-channel of Inception-V3 and CECED-channel of MobileNet-V3 (LCSC + CCI + CCM) for pneumonia identification. The proposed framework of the weighted fusion, as shown in Figure 8, clearly shows that the complimentary fusion of LBP, CLAHE, and CECED image features is capable of managing low-quality images in pneumonia diagnosis, attaining improved recognition accuracy on the CXR dataset. Figure 9 shows classification performance of all the weighted fusion channels including the single channels across all the evaluation metrics. It is obvious that our proposed model outweighs the other weighted fusion channels, as well as the single channels by a considerable margin.Although the computational time is slightly longer than the other weighted channels and the single models, yet it achieves the best classification performance, as depicted in Table 4.

### 4.5. Result of the Proposed Model

**Figure 10:**
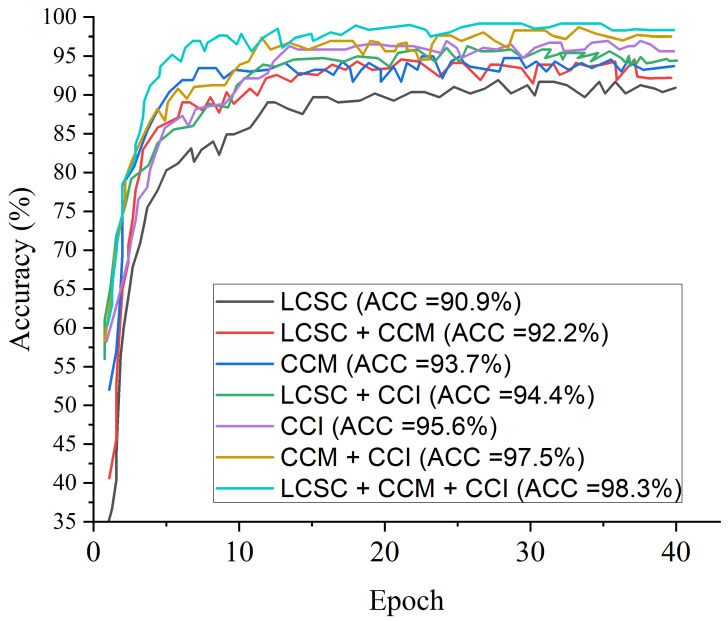
Accuracy curves for the proposed multi-channel in comparison with the single-channels and dual-channels.

**Figure 11:**
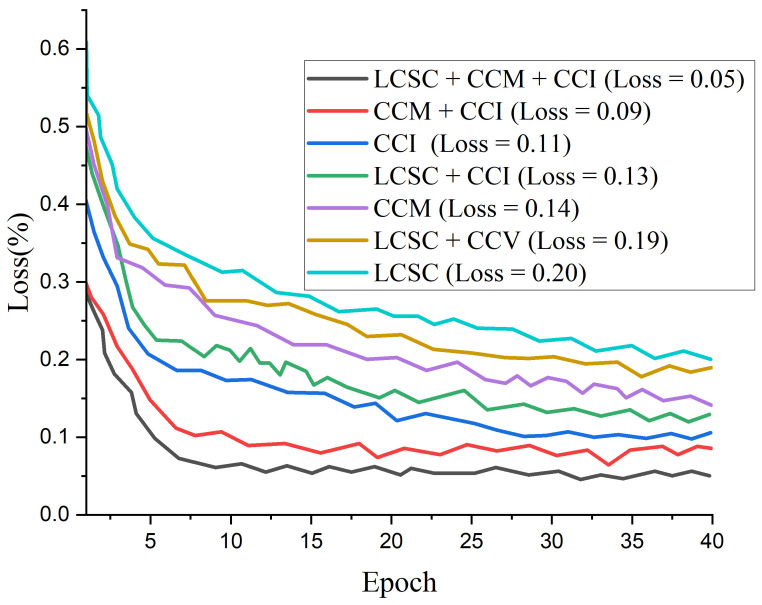
Loss curves for the proposed multi-channel in comparison with the single-channels and dual-channels.

**Figure 12:**
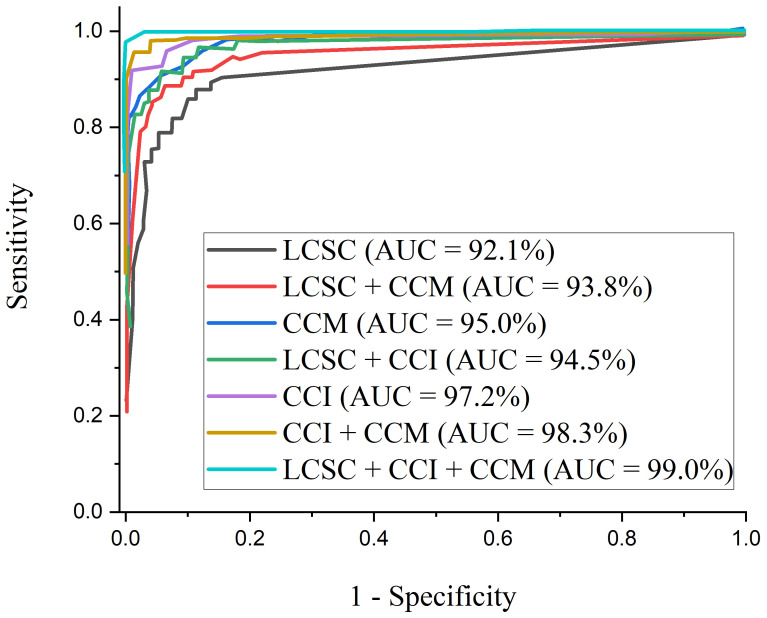
ROC curves for the proposed multi-channel in comparison with the single-channels and dual-channels.

Figure 10 presents the test accuracy curves for the proposed three-channel weighted fusion including the single- and dual-channel models. For the first 10 epochs, the accuracy rapidly increases to about 90% for the proposed model and then maintained gradual progression. Figure 11 depicts the loss curves which show the gradual loss reduction of the different models. Figure 12 presents the ROC-AUC curves for the single- and dual-channels, as well as the proposed weighted fusion deep learning schemes. The LCSC represents the LBP-based channel of shallow CNN for the identification of pneumonia with 92.1% AUC. The LCSC+CCM represents the weighted fusion of LBP-based channel of shallow CNN and the CECED-based channel of MobileNet-V3 with 93.8%. The CCM represents the CECED-based channel of MobileNet-V3 with 95.0% AUC. The LCSC+CCI represents the weighted fusion of LBP-based channel of shallow CNN and the CLAHE-based channel of Inception-V3 with 94.5% AUC. The CCI represents the CLAHE-based channel of Inception-V3 with 97.2% AUC. The CCI+CCM represents the weighted fusion of CLAHE-based channel of Inception-V3 and the CECED-based channel of MobileNet-V3 with 98.3%. The LCSC+CCI+CCM represents the weighted fusion of LBP-based channel of shallow CNN, the CLAHE-based channel of Inception-V3, and the CECED-based channel of MobileNet-V3 with 99.0% AUC.

**Figure 13:**
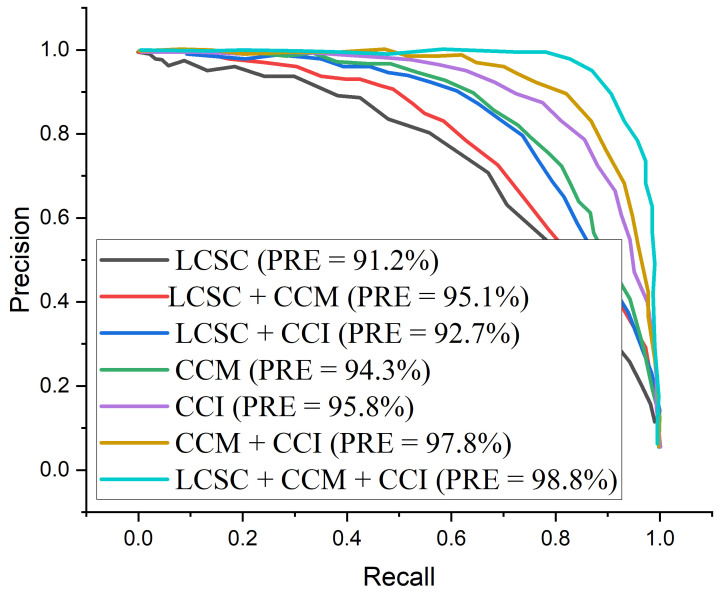
Precision–recall curves for the proposed multi-channel in comparison with the single-channels and dual-channels.

The proposed multi-channel weighted fusion model (LCSC + CCI + CCM) outperforms the single-based and dual-based channels on the pneumonia dataset, obtaining 99.0% AUC. The AUC of CECED-based channel of MobileNet-V3 is clearly higher than that of CLAHE-based channel of Inception-V3 and Shallow CNN of LBP-based channel, implying that CECED CXR images contribute more to pneumonia identification than CLAHE and LBP CXR images. The proposed weighted fusion model was further analyzed in terms of precision–recall curve, as shown in Figure 13. As the curve approaches the upper right hand corner of the graph, it is obvious that the proposed multi-channel weighted fusion model outweighs the other schemes, indicating that the model has high precision and high recall.

## 5. Discussion

In comparison to the single-based and dual-based channels, the proposed model’s performance in diagnosing pneumonia in CXR images from dataset obtained from various sources and combined has been demonstrated, and the identification result is given in Table 4. The proposed model can effectively differentiate distinct pneumonia from healthy CXR images, as evidenced by the above mentioned results. It is worth noting that the weighted fusion of LBP, CLAHE, and CECED CXR images result in a greater generalization ability for our proposed model.

We use the following terms to describe the channels as Shallow CNN for channel that employs LBP CXR images, inception-V3 for channel that uses CLAHE CXR images, and MobileNet-V3 for a channel that utilizes CECED CXR images. Table 4 shows the results of the identification for the different types of pneumonia. On all metrics, our proposed model surpasses the single-based and dual-based channels, with 98.3% accuracy, 98.9% sensitivity, and 99.2% specificity, 98.8% precision and 99% f1-score.

**Figure 14:**
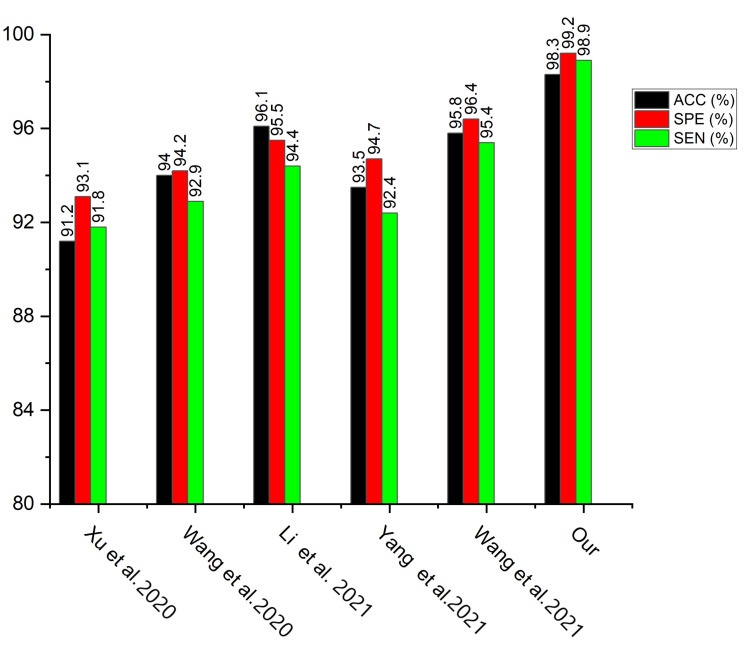
Performance evaluation for some selected state of the art models using the same dataset.

**Figure 15:**
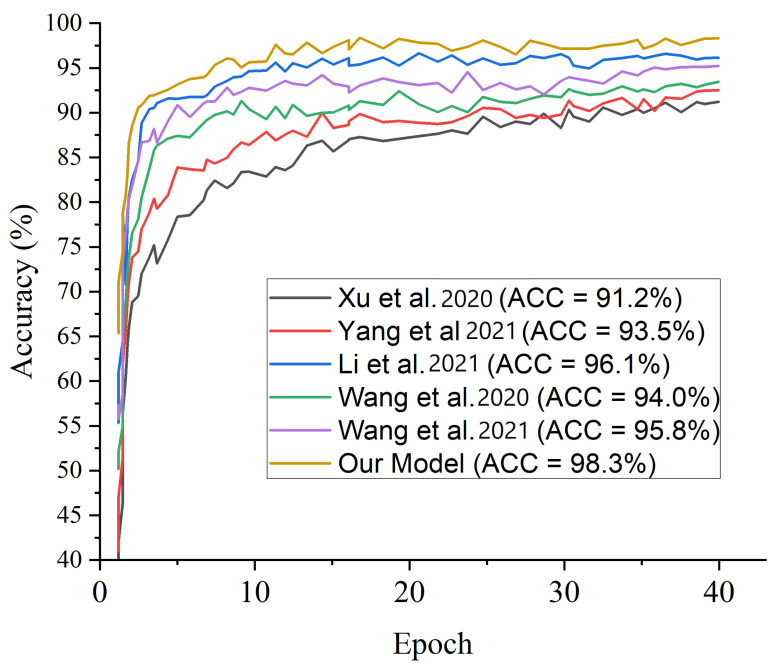
Accuracy performance for some selected state of the art models using the same dataset.

We conducted a comparison between our proposed strategy and some recently published pneumonia classification methods. According to Table 5, the proposed model obtained the maximum accuracy and sensitivity score of 98.3% and 98.9%, respectively, while Correa et al. [22] achieve the highest specificity score of 100% although their accuracy value was not reported. In terms of accuracy, the proposed model achieves the highest score of 98.3%, demonstrating its superiority in the identification of pneumonia. The complementing integration of several channels of CXR images gives our proposed technique a competitive advantage. It is worth noting that different deep learning models will perform differently depending on different circumstances. More so, we selected few recent state-of-the-art models and conducted a fair comparison using the same dataset as depicted in Table 6. Figure 14 presents the performance evaluation of the selected few state-of-the-art models across different matrices using the same dataset while Figure 15 depicts the accuracy performance of the selected few state-of-the-art models using the same dataset. We conducted an ablation study utilizing several transfer learning models pretrained on the ImageNet dataset in order to find the best performing pretrained model for our proposed weighted deep learning fusion architecture.

The results of the experiments employing the proposed scheme with several pretrained frameworks utilizing the same pneumonia dataset are presented in Table 7. According to the results of the experiments, Shallow CNN outperforms MobileNet-V3 and Inception-V3 in extracting features from LBP CXR images, reaching 90.9% accuracy, 92.3% sensitivity, and 93.1% specificity. In comparison to Shallow CNN, the MobileNet-V3 network performs better in extracting features from CECED CXR images sachieving 93.7% accuracy, 95.4% sensitivity, and 95.7% specificity. When compared to Shallow CNN and MobileNet-V3, Inception-V3 performs significantly better in extracting features from CLAHE CXR images, reaching reaching 95.6% accuracy, 94.9% sensitivity, and 96.2% specificity. It is essential to use the ROC curve to estimate overall accuracy and the precision–recall curve to measure the mean average precision of the proposed model when identifying sensitive conditions like pneumonia. The ROC curves for single- and dual-based channels, as well as the proposed model on the pneumonia dataset, are shown in Figure 12. Similarly, Figure 13 shows the precision–recall curve for single- and dual-based channels, as well as the proposed model.

Furthermore, many of the CXR images were blurry and lacking in details, which may have hampered the proposed model’s ability to extract and train relevant features. Interestingly, the benefits of utilizing LBP, CLAHE, and CECED preprocessing approaches to improve the low quality of CXR images, identify high representation details of the CXR images with visible trainable features. The proposed multi-channel weighted fusion performed admirably in identifying various kinds of pneumonia.

In terms of ROC and precision–recall curves, the proposed multi-channel weighted fusion model appears to outperform the other single and dual-based channels, particularly when dealing with low-quality CXR images. Our proposed model’s curve is closest to the upper left corner of the graph and has the largest area under curve, indicating that it has higher sensitivity and specificity according to the ROC graph in Figure 12. Similarly, our proposed model’s curve is closest to the graph’s upper right corner with the largest area, meaning that it has higher precision and sensitivity in Figure 13. More importantly, as explained above, the stated result in terms of Receiver Operating Characteristic (ROC) and precision–recall can aid expert radiologist in striking a balance between accuracy and precision.

Nevertheless, other methods have shown better performance for example, PneumoniaNet model [41]. PneumoniaNet model proposed by Alsharif et al. [41] uses CXR images to distinguish normal radiographic images from those with features consistent with viral or bacterial pneumonia in the pediatric group aged one–five years with a 99.72% accuracy, 99.74% sensitivity, 99.85% specificity, 99.7% precision, and 98.12% AUC. It achieved satisfactory performance because it combines the extracted feature of three subsequent convolution layers separated by ReLU and the batch normalization layer, whereas the general feature extraction uses only one convolution and batch normalization layer, hence permitting the CNN model to utilize the overall and marginal difference in CXR images.

The authors of PneumoniaNet seeks to optimize the information flow and gradient within the network, hence making the optimization of deep learning network tractable. This model improves the feature propagation, encourages feature re-useability and reduction in parameters. The distinguishing of this model and ResNet50 is that the latter learns from the reference input layers by using the output of the existing layer as the subsequent input layer as compared to the former, whereas comparing with VGG model, the PneumoniaNet model uses a deeper structure of small receptive subsequent 3×3 filters.

However, the authors did not consider the low quality of CXR images which is a major concern in real-life application. It is well known that data quality can be affected by factors such as noise, resolution, and artifacts, hence, using such data directly in an algorithm may affect model adaptability in medical application. A data preprocessing phase could help in eliminating or reducing the noise and increase the data quality, thereby improving model performance. The novelty of our methodology takes full advantage of the complementing integration of feature maps from multi-channels of data preprocessing technique in a weighted fusion manner, thereby enhancing the overall performance of our model.

### 5.1. Ablation Study

#### 5.1.1. Hyperparameter Tuning

We conducted another ablation study to examine if hyperparameter tuning may improve the performance of our proposed multi-channel model. The results of different optimizers at different learning rates and dropouts are shown in Table 8, Table 9 and Table 10. Our proposed multi-channel weighted fusion model obtains the best performance utilizing Adam optimizer with a learning rate of 0.0001 and dropout of 0.50, attaining 98.3% accuracy, according to Table 8. It is worth mentioning that the Adam optimizer is substantially more robust than the other optimizers (RMSProp and SGD) due to its computational efficiency.

#### 5.1.2. Raw X-ray Image Feature

**Figure 16:**
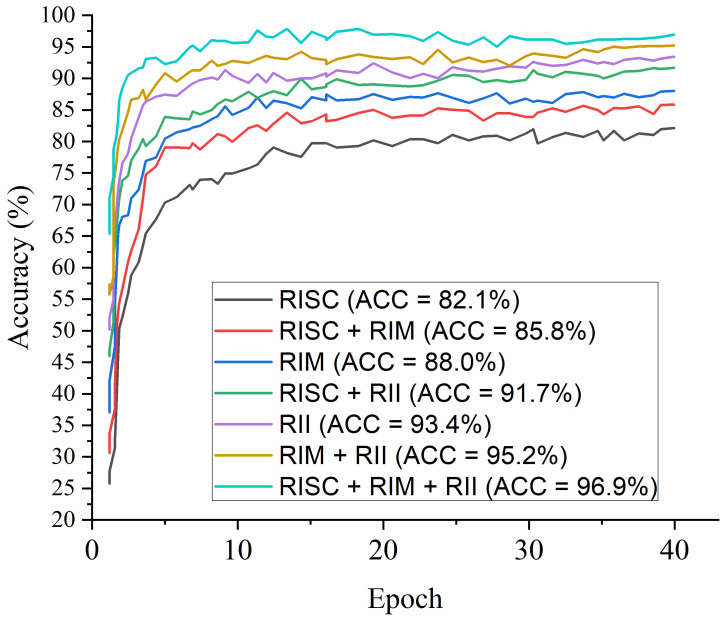
Accuracy results using the raw chest X-ray images.

**Figure 17:**
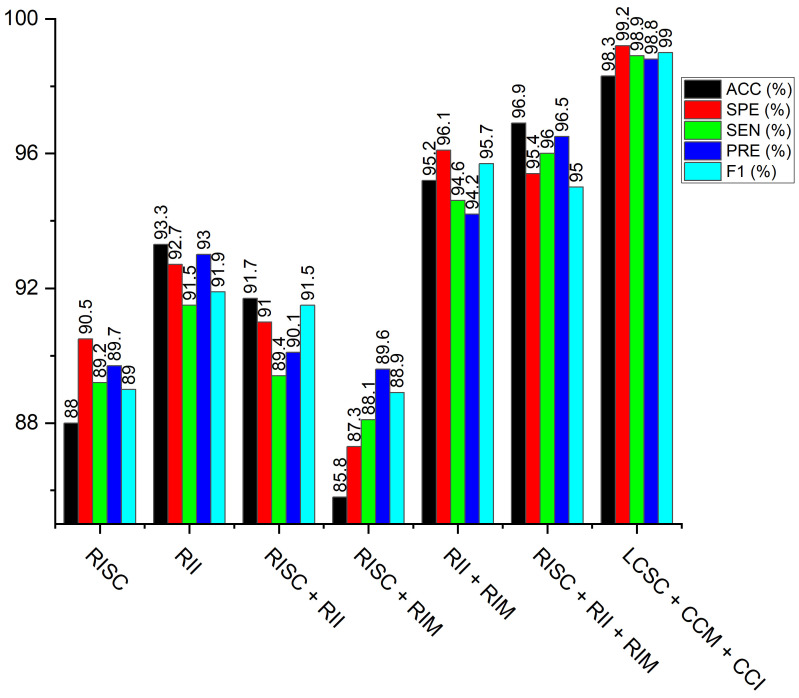
Performance evaluations using the raw chest X-ray images.

Further experiment is conducted to examine and validate the contribution of LBP, CECED and CLAHE features to model performance over raw X-ray image feature. Figure 16 shows the accuracy results using the raw chest X-ray images while Figure 17 depicts the performance evaluation of the models across different matrices using the raw chest X-ray images. Table 11 shows the performance of the various pre-trained models and shallow CNN using the preprocessed images including the raw X-ray image. To further validate the contribution of LBP, CECED, and CLAHE image features to the overall performance the proposed ensemble model, we conducted another experiment with the same selected pretrained models using the raw chest X-ray images, as depicted in Table 12. From all indication, the ensemble of LBP, CECED, and CLAHE yielded much better results compared to the ensemble of the raw chest X-ray image.

## 6. Conclusions

In this study, we proposed pneumonia identification method based on weighted fusion capable of processing LBP, CLAHE, and CECED CXR images simultaneously. We mentioned that the three channels are fused to complementary capture meaningful details from CXR images and achieved higher identification accuracy. The strategy of weighted fusion is utilized to take complete advantage of the visual features that have been captured from the different channels. The proposed weighted fusion model handles the problem of low-quality CXR images by fusing the weighted features generated from LBP, CLAHE, and CECED preprocessing stages. The shallow CNN, MobileNet-V3, and Inception-V3 models are employed to extract both healthy and non-healthy (COVID-19, virus, and bacteria pneumonia) features from LBP, CLAHE, and CECED CXR images.

Furthermore, these features are merged by utilizing the weighted fusion strategy in order to take advantage of the complementary lungs information. Softmax was introduced as the classifier to obtain the fused features. By fusing channels of complementary attributes in a weighted manner, our proposed model outweighs several state-of-the-art methods. The evaluation results show that the proposed model achieves better performance with accuracy of 98.3%, sensitivity of 98.9%, specificity of 99.2%, precision of 98.8% and f1-score of 99.0% than just using the single- and dual-channels. From the comparative results of the other established methods, it is confirmed that the proposed weighted fusion model achieved state-of-the-art identification accuracy, which makes it robust and efficient identification solution for pneumonia based on low quality CXR images. These results could effectively assist the expert radiologist to diagnose what type of pneumonia disease is present in a patient’s lungs while saving screening time.

## Figures and Tables

**Table 1:** Summary of the Related Works.

Authors	Year	Mode of Imaging	DL Techniques	Classification Task	Evaluation Results
Cicero et al. [19]	2017	X-ray Image	GoogLeNet is utilized to classify two classes - normal and abnormal images	Binary class	For normal class: SEN = 91%, SPE = 91%, and UC = 96.4% For abnormal class: SEN (within 74% to 91%), SPE (within 75% to 91%), and AUC (within 85% to 96.2%)
Guendel et al. [20]	2018	X-ray Image	Used location-aware dense networks technique to identify anomalies in chest X-rays	Multiple class	PLCO dataset, mean AUC = 87.4%, Chest X-ray 14 dataset, mean AUC = 84.1%
Rajaraman et al. [21]	2018	X-ray Image	A modified VGG16 is employed for the identification and detection of viral and bacterial pneumonia	Binary class	ACC (within 91.8% to 96.2%)
Correa et al. [22]	2018	Ultrasound Image	Detection of pneumonia using 3 layers feed-forward neural network	Binary class	SEN = 90.9% SPE = 100%
Ke et al. [23]	2019	X-ray Image	Detection of lung diseases using an approach called neuroheuristic	Multiple class	Sensitivity = 84.22%, Accuracy = 79.06%, Specificity = 66.7%
Saraiva et al. [24]	2019	X-ray Image	A CNN model was applied on a dataset of 5863 images and cross-validation was used for the validation of the model	Binary class	Accuracy = 95.30%
Sirazitdinov et al. [25]	2019	X-ray Image	An emsemble of RetinaNet and Mask RCNN was applied	Binary class	Precision = 75.0%, Recall = 79%, F1-score = 77.0%
Liang and Zheng [26]	2020	X-ray Image	A modified 49 convolutional and 2 fully connected layer of a CNN model was used for the classification of children’s lung regions	Binary class	F1-score = 92.7%
Apostolopoulos et al. [27]	2020	X-ray Image	Different fine-tuning approaches were evaluated for the automatic detection of pneumonia	Binary class	VGG19 has the highest value of: Sensitivity = 92.85%, Specificity = 98.75%, Accuracy = 98.75%
Xu et al. [28]	2020	X-ray Image	Multiple CNN models were compared in order to categorize the classes of CT scans	Multiple class	Accuracy = 86.7%
Habib et al. [29]	2020	X-ray Image	Detection of pneumonia using an ensemble of VGG-19 and CheXNet for the extraction of features and random forest as the classifier	Binary class	Accuracy = 98.93%
Chouhan et al. [30]	2020	X-ray Image	A transfer learning technique is applied for the detection of pneumonia	Binary class	Accuracy = 96.4% Sensitivity 99.0%
El Asnaoui et al. [31]	2020	X-ray Image	A fine-tuned of eight different models for the detection and classification of pneumonia	Binary class	Highest accuracy is the fine-tubed ResNet50 (>96%)
El Asnaoui et al. [32]	2020	X-ray Image	A comparative findings of seven DL models for the classification and detection of pneumonia (including COVID-19)	Multiple class	Accuracy Evaluations: InceptionResNet-V2 = 92.18%, DenseNet201 = 88.09%

**Table 2:** Description of the Dataset.

Dataset	Pneumonia Category	Value	Selected Amount Used
Kaggle database of RSNA [33]	Bacterial	3029	1000
	Viral	2983	1000
	Normal	8851	1000
Rahman et al. [34]	COVID-19	3616	1000

**Table 3:** Parameter for the modified MobileNet-V3. bneck represents bottleneck convolution, SE depicts whether there is a Squeeze-and-Excite in that block, NL represents the type of non-linearity utilized, HS represents h-swish, RE denotes ReLU and S represents stride.

Input	Operator	Expansion Size	Output	SE	NL	Stride
224 × 224 × 3	Conv2d, 3 × 3	-	16	No	HS	2
112 × 112 × 16	bneck, 3 × 3	16	16	Yes	RE	2
56 × 56 × 16	bneck, 3 × 3	72	24	No	RE	2
28 × 28 × 24	bneck, 3 × 3	86	24	No	RE	1
28 × 28 × 24	bneck, 5 × 5	96	40	Yes	HS	2
14 × 14 × 40	bneck, 5 × 5	240	40	Yes	HS	1
14 × 14 × 40	bneck, 5 × 5	240	40	Yes	HS	1
14 × 14 × 40	bneck, 5 × 5	120	48	Yes	HS	1
14 × 14 × 48	bneck, 5 × 5	144	48	Yes	HS	1
7 × 7 × 96	bneck, 5 × 5	288	96	Yes	HS	2
7 × 7 × 96	bneck, 5 × 5	576	96	Yes	HS	1
7 × 7 × 96	bneck, 5 × 5	576	96	Yes	HS	1
7 × 7 × 256	Conv2d, 1 × 1	-	256	Yes	HS	1
1 × 1 × 256	Avg pool, 7 × 7	-	-	No	-	1
1 × 1 × 512	Conv2d, 1 × 1	-	512	No	HS	1

**Table 4:** Comparison of our proposed model with single channels and dual channels.

Model	ACC (%)	SEN (%)	SPE (%)	PRE (%)	F1-s (%)	Time (min)
LBP-Channel Shallow CNN (LCSC)	90.9	92.3	93.1	91.2	92.7	3.2
CECED-Channel MobileNet-V3 (CCM)	93.7	95.4	95.7	94.3	95.5	18.6
CLAHE-Channel Inception-V3 (CCI)	95.6	94.9	96.2	95.8	95.3	21.8
LBP-Channel Shallow CNN + CECED-channel MobileNet-V3 (LCSC + CCM)	92.2	93.7	94.5	92.7	94.3	23.4
LBP-Channel Shallow CNN + CLAHE-channel Inception-V3 (LCSC + CCI)	94.4	95.5	96.8	95.1	96.6	22.7
CLAHE-Channel inception-V3 + CECED-channel MobileNet-V3 (CCI + CCM)	97.5	97.3	98.3	97.8	98.1	26.8
LBP-Channel Shallow CNN + CLAHE-channel Inception-V3 + CECED-channel MobileNet-V3 (LCSC + CCI + CCM)	98.3	98.9	99.2	98.8	99.0	30.3

**Table 5:** Result comparison of our proposed model with state-of-the-art methods for pneumonia classification.

Authors	ACC (%)	SEN (%)	SPE (%)
Cicero et al. [19]	91.0	91.0	91.0
Correa et al. [22]	-	90.9	100.0
Apostolopoulos et al. [27]	98.0	92.9	98.8
Xu et al. [28]	86.7	86.9	-
Habib et al. [29]	98.93	-	-
Chouchan et al. [30]	96.4	99.6	-
Yamaç et al. [35]	86.5	79.2	90.7
Wang et al. [36]	93.3	90.7	95.5
Li et al. [37]	96.9	97.8	94.9
J.K. K. Singh and A. Singh [38]	95.8	96.1	95.7
Yang et al. [39]	88.4	64.7	92.9
Wang et al. [40]	94.5	94.7	97.3
Alsharif et al. [41]	99.7	99.7	99.8
Alqudah et al. [42]	93.9	93.2	96.6
Alquran et al. [43]	93.1	92.9	96.4
Masad et al. [44]	98.9	98.3	99.2
Our Model	98.3	98.9	99.2

**Table 6:** Comparison table for the selected state of the art models using the same dataset.

Model	ACC (%)	SEN (%)	SPE (%)
Xu et al. [28]	91.2	91.8	93.1
Wang et al. [36]	94.0	92.9	94.2
Li et al. [37]	96.1	94.4	95.5
Yang et al. [39]	93.5	92.4	94.7
Wang et al. [40]	95.8	95.4	96.4
Our Model	98.3	98.9	99.2

**Table 7:** Results obtained on our dataset using different pretrained models on our proposed model.

Model	LBP-Based Channel			CECED-Based Channel			CLAHE-Based Channel		
	ACC (%)	SEN (%)	SPE (%)	ACC (%)	SEN (%)	SPE (%)	ACC (%)	SEN (%)	SPE (%)
AlexNet	89.2	91.4	92.6	89.3	87.5	90.2	92.5	94.3	93.7
VGG-16	88.9	90.7	91.4	90.9	90.1	91.8	91.4	92.8	91.9
ResNet-152	84.6	86.2	87.9	91.4	92.3	93.1	87.8	88.1	87.6
MobileNet-V3	87.7	89.4	90.5	93.7	95.4	95.7	90.4	91.6	90.8
DenseNet-121	85.3	87.1	88.7	92.8	92.8	93.3	88.4	89.2	88.7
Inception-V3	86.3	88.6	89.4	93.1	91.5	93.7	95.6	94.9	96.2
Shallow CNN	90.9	92.3	93.1	87.2	86.1	88.4	85.9	86.2	85.7

**Table 8:** Performance evaluation of our proposed model based on different hyperparameter tuning on our dataset with Adam optimizer.

Hyperparameters	(LCSC + Adam)	(CCI + Adam)	(CCM + Adam)	(LCSC + CCI + Adam)	(LCSC + CCM + Adam)	(CCI + CCM + Adam)	(LCSC + CCI + CCM + Adam)
	Accuracy (%)	Accuracy (%)	Accuracy (%)	Accuracy (%)	Accuracy (%)	Accuracy (%)	Accuracy (%)
LR (0.1) + Dropout (0.25)	87.5	81.6	89.6	87.3	85.6	90.2	88.1
LR (0.1) + Dropout (0.50)	86.9	87.3	90.7	89.6	87.4	89.5	87.6
LR (0.1) + Dropout (0.75)	83.6	82.6	87.9	89.9	89.3	91.7	89.2
LR (0.01) + Dropout (0.25)	81.4	85.1	91.1	90.4	91.7	90.4	87.1
LR (0.01) + Dropout (0.50)	89.8	84.9	86.3	91.1	90.8	85.5	88.4
LR (0.01) + Dropout (0.75)	84.7	90.7	92.8	85.7	89.2	87.8	89.7
LR (0.001) + Dropout (0.25)	82.2	95.6	88.2	84.6	92.1	92.1	91.5
LR (0.001) + Dropout (0.50)	80.7	91.3	93.4	86.5	89.7	94.7	92.6
LR (0.001) + Dropout (0.75)	88.3	92.7	93.7	91.4	90.8	89.6	93.8
LR (0.0001) + Dropout (0.25)	85.9	83.4	85.6	87.9	92.4	96.3	97.4
LR (0.0001) + Dropout (0.50)	90.9	80.6	87.1	94.4	92.2	97.5	98.3
LR (0.0001) + Dropout (0.75)	79.5	86.2	88.9	89.8	93.3	95.7	95.9

**Table 9:** Performance evaluation of our proposed model based on different hyperparameter tuning on our dataset with RMSProp optimizer.

Hyperparameters	(LCSC + RMSProp)	(CCI + RMSProp)	(CCM + RMSProp)	(LCSC + CCI + RMSProp)	(LCSC + CCM + RMSProp)	(CCI + CCM + RMSProp)	(LCSC + CCI + CCM + RMSProp)
	Accuracy (%)	Accuracy (%)	Accuracy (%)	Accuracy (%)	Accuracy (%)	Accuracy (%)	Accuracy (%)
LR (0.1) + Dropout (0.25)	88.5	89.1	88.9	87.6	90.7	89.7	90.1
LR (0.1) + Dropout (0.50)	89.7	87.4	89.5	87.2	89.5	90.3	91.3
LR (0.1) + Dropout (0.75)	86.3	90.7	88.1	89.3	89.6	91.6	89.8
LR (0.01) + Dropout (0.25)	87.7	88.2	89.6	88.4	88.4	92.5	91.6
LR (0.01) + Dropout (0.50)	81.4	91.5	91.2	90.6	90.9	90.9	93.4
LR (0.01) + Dropout (0.75)	81.1	89.8	90.4	89.7	91.6	92.1	92.8
LR (0.001) + Dropout (0.25)	83.8	92.3	92.2	88.9	90.1	91.4	94.4
LR (0.001) + Dropout (0.50)	86.5	89.6	94.3	87.3	93.7	93.7	96.5
LR (0.001) + Dropout (0.75)	84.2	90.9	93.5	90.4	92.4	95.3	95.9
LR (0.0001) + Dropout (0.25)	85.9	91.1	94.7	91.8	93.6	94.5	97.4
LR (0.0001) + Dropout (0.50)	88.6	89.5	93.9	90.6	92.9	93.9	96.2
LR (0.0001) + Dropout (0.75)	85.3	88.9	94.6	89.7	91.5	92.6	95.5

**Table 10:** Performance evaluation of our proposed model based on different hyperparameter tuning on our dataset with SGD optimizer.

Hyperparameters	(LCSC + SGD)	(CCI + SGD)	(CCM + SGD)	(LCSC + CCI + SGD)	(LCSC + CCM + SGD)	(CCI + CCM + SGD)	(LCSC + CCI + CCM + SGD)
	Accuracy (%)	Accuracy (%)	Accuracy (%)	Accuracy (%)	Accuracy (%)	Accuracy (%)	Accuracy (%)
LR (0.1) + Dropout (0.25)	87.2	88.8	87.7	86.1	87.1	90.8	89.7
LR (0.1) + Dropout (0.50)	85.5	89.3	88.1	88.5	89.6	89.1	90.5
LR (0.1) + Dropout (0.75)	87.9	97.5	89.5	87.9	88.2	90.9	91.6
LR (0.01) + Dropout (0.25)	89.1	90.9	91.9	89.7	89.7	91.7	89.1
LR (0.01) + Dropout (0.50)	82.3	92.3	90.3	91.5	91.4	92.3	91.3
LR (0.01) + Dropout (0.75)	83.6	88.7	89.5	90.3	90.1	90.8	93.5
LR (0.001) + Dropout (0.25)	84.9	91.9	91.7	89.1	91.2	92.5	92.7
LR (0.001) + Dropout (0.50)	85.7	90.4	92.4	90.5	92.5	91.6	95.9
LR (0.001) + Dropout (0.75)	83.5	91.6	94.1	91.9	93.8	94.9	94.4
LR (0.0001) + Dropout (0.25)	86.3	89.3	93.3	89.7	92.9	95.3	96.2
LR (0.0001) + Dropout (0.50)	87.1	90.8	94.6	91.5	93.6	92.5	95.6
LR (0.0001) + Dropout (0.75)	88.8	89.2	93.0	90.3	92.3	93.7	96.8

**Table 11:** Performance evaluation of the proposed model on the single and ensemble models using raw CXR image.

Model	ACC (%)	SEN (%)	SPE (%)	PRE (%)	F1 (%)
Raw image Shallow CNN (RISC)	82.1	83.6	80.8	82.9	83.3
Raw image MobileNet-V3 (RIM)	88.0	89.2	90.5	89.7	89.0
Raw image Inception-V3 (RII)	93.34	91.5	92.7	93.0	91.9
Raw image Shallow CNN + Raw image Inception-V3 (RISC + RII)	91.7	89.4	91.0	90.1	91.5
Raw image Shallow CNN + Raw image MobileNet-V3 (RISC + RIM)	85.8	86.1	87.3	89.6	88.9
Raw image Inception-V3 + Raw image MobileNet-V3 (RII + RIM)	95.2	94.6	96.1	94.2	95.7
Raw image Shallow CNN + Raw image Inception-V3 + Raw image MobileNet-V3 (RISC + RII + RIM)	96.9	96.0	95.4	96.5	95.0

**Table 12:** Performance results obtained using the raw chest X-ray images on different pretrained models on our proposed model.

Model	Raw Image		
	ACC (%)	SEN (%)	SPE (%)
AlexNet	90.9	89.1	91.0
VGG-16	89.6	90.3	89.2
ResNet-152	90.2	88.5	89.0
MobileNet-V3	88.0	89.2	90.5
DenseNet-121	87.7	89.1	88.3
Inception-V3	93.3	91.5	92.7
Shallow CNN	82.1	83.6	80.8

## Data Availability

The dataset used in this study can be found in references [33,34].
